# In Vitro Activity of Several Essential Oils Extracted from Aromatic Plants against *Ascosphaera apis*

**DOI:** 10.3390/vetsci8050080

**Published:** 2021-05-10

**Authors:** Michelina Pusceddu, Ignazio Floris, Nicoletta P. Mangia, Alberto Angioni, Alberto Satta

**Affiliations:** 1 Department of Agricultural Sciences, University of Sassari, 07100 Sassari, Italy; mpusceddu@uniss.it (M.P.); ifloris@uniss.it (I.F.); nmangia@uniss.it (N.P.M.); 2 Department of Life and Environmental Sciences, University of Cagliari, 09124 Cagliari, Italy; aangioni@unica.it

**Keywords:** chalkbrood, honey bee diseases, biological control, aromatic plants

## Abstract

The use of natural substances such as essentials oils against bee pathogens is of great interest as an alternative to traditional methods based on synthetic compounds like antibiotics and fungicides, in order to minimize the risk of having toxic residues in hive products and to prevent the development of resistance phenomena. This study evaluated the inhibitory, fungicidal and sporicidal activity of ten essential oils extracted from aromatic plants against *Ascosphaera apis*, the etiological agent of chalkbrood, an invasive honey bee mycosis. The most effective essential oils were *Thymus herba-barona, Thymus capitatus* and *Cinnamomum zeylanicum*, which showed values of minimum fungicidal concentration and minimum sporicidal concentration ranging from 200 to 400 ppm. Carvacrol was the main component of *Thymus capitatus* and *Thymus herba-barona* oils, whereas cinnamic aldehyde prevailed in *Cinnamomum* *zeylanicum* oil. Further in-apiary studies will allow the evaluation of side effects on bees and residues in hive products.

## 1. Introduction

All the identified species of fungi belonging to the genus *Ascosphaera* (Ascomycota: Plectomycetes; Ascosphaearales) have been detected in bees [1,2]. The fungus *Ascosphaera apis* (Maassen ex Claussen) Olive and Spiltoir is the etiological agent of the invasive honey bee mycosis named chalkbrood [3]. It is a heterothallic organism that sporulates when mycelia of two different strains of the opposite sex touch each other and fruiting bodies are formed [4]. The disease transmission occurs through the ingestion of spores from contaminated food by the young bee larvae [5]. After infection, the larvae reduce their food consumption quickly until they stop eating [5]. Bee larvae in the fifth instar are the most sensitive to the disease, as they have favorable environmental conditions in their gut for spore germination [6,7,8]. Once in the larval gut, the spores are activated by CO_2_ obtained from the cells [9]. Then, they can germinate in the lumen, producing a mycelium which pierces the larval cuticle [9]. In this phase, the larvae look like tiny pieces of chalk or “mummies”, which gives the name of chalkbrood to the disease [7,10]. As the disease progresses, the larvae become mummified, changing from white to a dark gray or black color due to the presence of spores on the larval cuticle [10,11].

This fungal disease occurs widely in temperate regions and is most prevalent in the spring when there is an increase in the brood in the hive [12]. Fungal spores confined in wax and food stored inside the beehive are very resistant and remain infective for many years, providing a continuous source of infection [7,11]. The spread of *A. apis* infection in apiary can also be facilitated by its interaction with other pathogens or parasites such as the ectoparasite *Varroa destructor* [13,14]. In fact, the body surface of mites can become contaminated with fungal spores [15,16], but doubts remain as to whether these can cause infection, since many spores would be required to contaminate the larval food and be ingested by the developing honey bee brood. This would preclude effective direct transmission of *A. apis* by mites. In addition, other factors, such as the genetic strain of the pathogen or the host, can influence the incidence and severity of the disease [17]. Although chalkbrood rarely leads to the collapse of the colonies, it can weaken the hives, with a consequent reduction in honey production and considerable economic losses [18]. The incidence of chalkbrood may be on the rise around the world [19].

Many different chemical compounds have been tested in order to control the chalkbrood disease [10,20,21,22], but no effective drugs against this fungal disease are currently available. When the colonies are infected, only a temporary reduction of symptoms has been achieved [22]. In fact, it is extremely difficult to eradicate this disease, because chemical products do not kill the spores, which are very persistent in the hive environment [11]. In addition, synthetic antifungal agents have several negative effects such as reductions in bee vitality in various life stages, an increase in resistance development and the contamination of hive products [23].

The use of natural substances in apiculture is of great interest because of the reduced risk of leaving harmful residues for human consumers [12,24]. One of the most tested groups of natural compounds against different honey bee diseases is represented by essential oils (EOs) [25], the antifungal activity of which is probably due to phenols and terpenic compounds [23]. The interest in testing essential oils as an alternative control strategy against *A. apis* has grown over the past years [5,12,23,26,27,28,29,30,31].

Essential oils are hydrophobic liquids containing bioactive volatile compounds with high chemical variability [32], which complicates the study of their pharmacological power. In this work, we evaluated the in vitro activity of 10 essential oils from aromatic plants, most of them typical of the Mediterranean region, against *A. apis*, using the agar diffusion method. In addition, bioassays were performed to assess the minimum inhibitory concentration (MIC), the minimum fungicidal concentration (MFC) and the minimum sporicidal concentration (MSC) values (ppm) of the four essential oils, which showed inhibition haloes greater than that of nystatin (the positive control). They were also chemically characterized by means of gas chromatography–flame ionization detector (GC-FID) and gas chromatography–mass spectrometry analysis (GC-MS).

## 2. Materials and Methods

### 2.1. Ascosphaera apis Culture and Inoculum Preparation

The reference strain *Ascosphaera apis* 3116 used in this study was provided by the DMSZ collection (German Collection of Microorganism and Cell Cultures GmbH). The strain was stored at 4 °C until microbiological tests. Fresh culture of *A. apis* was obtained by growing the fungus on a Sabouraud dextrose agar (SDA; Oxoid, Milan, Italy) at 30 °C for 5 days in aerobic conditions, used as a stock plate. Fungal spores were obtained by exposing *A. apis* culture in Sabouraud dextrose broth (SDB) at a high level of CO_2_ and at 37 °C for 4 h and then at 20 °C for 72 h. After microscopic enumeration with a cell-counting hemocytometer (Neubauer chamber; Merck, Milan, Italy), the spore concentration was adjusted to about 1×10^5^ spores/mL before final inoculation.

### 2.2. Plant Collection and Essential Oil (EO) Preparation

The following officinal plants were collected in 2017 during their flowering period in Sardinia (Italy): *Thymus capitatus* (L.) Hofimans and Link, *Thymus herba-barona* Loisel (endemic to Sardinia and Corsica), *Rosmarinus officinalis* (L.), *Myrtus communis* L., *Eucalyptus globulus* Labill., *Salvia desoleana* Atzei and Picci (endemic to Sardinia), *Salvia officinalis* (L.), and *Helichrysum italicum* subs. *microphyllum* G. Don. (endemic to Sardinia, Corsica, Balearic Islands and Crete). Moreover, we also tested two commercial essential oils provided by Cruciani (Rome, Italy), *Cinnamomum zeylanicum* and *Rosmarinus officinalis*. The former is known for its sporicidal activity against *Paenibacillus larvae*, the agent of the American foulbrood [33]. Flowering tops and stems of each of the eight species were collected from the plants at sunrise, stored immediately at a temperature below 24 °C and brought to the laboratory within 6 h after harvest for analysis. The distillation process was performed on homogeneous samples (same weight), without any pre-processing, in a 50-L steel steam distiller (Albrigi-Stallavena, Verona, Italy) with a recycling system for about 2 h. At the end of distillation, the EOs, separated from water by decantation, were recovered directly from the distillate and dried over anhydrous Na_2_SO_4_ according to the method of Angioni et al. [33]. The oils were stored at 4 °C in the dark until use.

### 2.3. Antifungal Test of Essential Oils (EOs)

#### 2.3.1. Agar Diffusion Method

A first screening to establish the susceptibility of *A. apis* to EOs was carried out using the paper disc agar diffusion (PDD) method. From an SDA agar plate, a mycelium disc of 6 mm in diameter was taken, dissolved in Ringer solution (Oxoid) and vortexed for 5 min. Subsequently, 1 mL of the fungal suspension was overlaid on the SDA plate. After plate solidification, sterile filter paper discs (6 mm in diameter, Oxoid, Milan, Italy), saturated with each pure essential oil (*n* = 10), were placed over the plate surface (PDD method). The cultures were then incubated at 30 °C for 5 days. Blank discs and synthetic antifungal nystatin were used as negative and positive controls, respectively. The diameter (mm) of fungal growth inhibition was measured (disk diameter included). An EO activity level higher than 20 mm was classified as high activity. All the experiments were carried out in triplicate for each essential oil.

#### 2.3.2. Microdilution Method

In the second stage of the experiment, minimum inhibitory concentration (MIC), minimum fungicidal concentration (MFC) and minimum sporicidal concentration (MSC) values of the four essential oils which showed inhibition haloes greater than that of nystatin (the positive control), i.e., with a minimum halo diameter > 20 mm, were determined using the microdilution method based on standard CLSI (2008) guidelines [34] with minor modifications.

Briefly, MIC determination was performed on a 96-well microtiter plate (Merk, Milan, Italy) containing SDB medium. The EOs were first diluted to obtain the highest concentration of 500 ppm, which was further diluted to obtain the following concentrations: 400, 300, 200, 100 and 50 ppm. The highest oil concentration used in our bioassays was based on previous tests evaluating the possible side effects of these oils on adult bees [35]. Each well was inoculated with a fungal suspension (obtained as described above) in such a volume as to obtain a concentration of about 106 CFU/mL. Then, the microplates were kept at 30 °C for 5 days. The lowest concentration of the EOs in which there was no visible growth (i.e., the absence of turbidity in the well) was taken as the MIC [36].

Furthermore, in order to establish the MFC, the subcultures taken from the MIC wells where no visible growth was recorded were streaked on an SDA plate. After incubation at 30 °C for 5 days in aerobic conditions, the fungal viable growth was evaluated. The lowest concentration of the EOs in which there was no fungal growth was taken as the MFC. Finally, to evaluate the minimum sporicidal concentration (MSC), the same method was used; the microplates containing the EOs at different concentrations were inoculated with an aliquot of the fungal spores’ culture (described above) and incubated at 30 °C for 5 days. An aliquot from the well, in which there was no visible growth, was taken and inoculated on an SDA plate. After incubation time, the lowest concentration of the EOs in which there was no viable growth on the SDA plate was taken as the MSC. All trials were made with three replications for each EO tested. Because the results were homogeneous in all three replicates, the values of MIC, MFC and MSC were reported in the table without a measure of variability. SDB medium without essential oils was used as a control.

### 2.4. Chemical Composition

The analytical standards (97%) used for confirmation and quantification analysis were purchased from Extrasynthese (Genay, France) and Sigma Aldrich (Milan, Italy). 2,6-dimethylphenol was used as an internal standard (99.8%; Sigma Aldrich, Milan, Italy). Solutions of 1% (*w*/*v*) oil were prepared in hexane for gas chromatography (GC) analysis. Quali-quantitative analyses of the EOs were performed in triplicate, obtained from independent distillation experiments.

The chemical composition of the oils was assessed using a gas chromatography–flame ionization detector (GC-FID) and gas chromatography–mass spectrometry (GC-MS) analysis. An HROC 5300 Mega series gas-chromatograph, coupled with an FID (Carlo Erba, Milan, Italy), was used for quantitative analysis, whereas a Hewlett Packard 5890 series II gas-chromatograph (Hewlett Packard, Avondale, PA), equipped with a HP 5971 A (70 eV) mass selective detector (MSD) in manual injection mode, a split-splitless injector and an MS ChemStation HP v. C.00.07, was used for peak identification and confirmation. The column was a fused silica capillary DB-5MS (5% phenylmethylpolysiloxane, 30 m × 0.25 mm id, 0.25 µm film thickness) (J&W Scientific Fisons, Folsom, CA, USA). The injector and the interface were operated at 200 °C and 280 °C, respectively. The oven temperature was programmed as follows: from 60 °C to 180 °C (3 °C/min) and isothermally held for 15 min. Helium was the carrier gas at 0.9 mL/min, and the sample (1 µL) was injected in split mode (1:20). MS conditions were as follows: ionization voltage, 70 eV; scan rate, 1.6 scan/s; mass range, 40–500; and ion source temperature, 180 °C. The identification of the essential oil compounds was based on comparison of the retention times with those of authentic samples, comparing their linear retention index (LRI) relative to the series of n-hydrocarbons, and on computer matching against mass spectrum commercial libraries (NIST 98 and ADAMS) and a homemade library built up from pure substances and components of known oils and MS literature data.

### 2.5. Statistical Analysis

ANOVA was used to analyze the inhibition halo data after logarithmic transformation (log (1 + x)) to reduce the heterogeneity of the variance, checked using the Shapiro–Wilk test. When significant differences were detected, means were separated using the Tukey post-hoc test. Table 1 shows the untransformed values. All tests were carried out using R software [37] implemented with the agricolae library.

## 3. Results

Among the 10 pure essential oils tested against *A. apis* using the PDD method, the most effective were *T. herba-barona* and *T. capitatus*, which fully blocked the growth of the fungus (Table 1). On average, the essential oils from *H. italicum* and *C. zeylanicum* produced an inhibition halo of 40.0 and 24.8 mm, respectively (Table 1). A lower inhibiting effect was observed for the oils from *R. officinalis* (commercial and non-commercial), *E. globulus* and *M. communis* plants, which showed an inhibition halo smaller than 16 mm. *S. desoleana* and *S. officinalis* did not show any antimicrobial activity, as observed in the control plate. Nystatin showed a mean inhibition diameter of 17.0 mm, similar to the commercial *Rosmarinus* and lower than *Thymus*, *Helichrysum* and *C. zeylanicum* (Table 1).

Based on the agar diffusion results, in which pure essential oils were employed, the microdilution assay and characterization analysis were conducted only on the four essential oils that showed the highest activity (inhibition halo diameters larger than 20 mm). The assays on the vegetative forms performed in Sabouraud broth showed an inhibitory effect (MIC) on the growth of *A. apis* at 100 ppm for *T. capitatus* and *T. herba-barona* oils and 200 ppm for *Cinnamomum* oil, whereas *Helichrysum* did not show any inhibitory activity even at the highest concentration (500 ppm) (Table 2). In the assays carried out in Sabouraud dextrose agar, *Cinnamomum* essential oil showed an MFC at 200 ppm, whereas both thyme oils demonstrated an MFC at 300 ppm (Table 2). In contrast, *Helichrysum* essential oil did not show any inhibition activity at any of the concentrations tested in this type of assay (Table 2). The inhibitory test carried out on the *A. apis* spores showed an overall high resistance of these forms to the essential oils. Only MSC values of 400 ppm showed a sporicidal effect for both *Thymus* oils and the *Cinnamomum* oil, whereas *Helichrysum* required an MSC of 500 ppm (Table 2). In all bioassays performed, we observed a regular growth of the fungus *A. apis* in the control treatment.

The GC-MS analysis allowed the identification of 20 compounds in thyme samples. The main component of thyme essential oils was carvacrol (68.0% for *T. capitatus* and 60.0% for *T. herba-barona*), followed by γ-terpinene, p-cymene, β-cariophyllene and β-myrcene. *Cinnamomum* essential oil showed 22 compounds, but it was constituted mainly of cinnamic aldehyde (79.3%) and eugenol (11.9%), with the amounts other compounds below 0.5%, except for α-pinene (1.8%) and α-thujene (1.1%). *Helichrysum* essential oil showed 24 identified compounds, with the main compounds derived from the nerol series. Neryl acetate was the main compound, accounting for 51.6%, followed by nerol (8.22%) and neryl propionate (5.51%). β-Eudesmol, γ-curcumene, limonene, α-terpineol, linalyl acetate and α-eudesmol showed values ranging between 1.06% and 2.27%, whereas curcumene and linalool showed values below 5% (Table 3).

## 4. Discussion

Among the various essential oils tested in this study, mostly from Mediterranean aromatic plants, the main finding was the higher efficacy of *Thymus capitatus*, *Thymus herba-barona*, *Cinnamomum zeylanicum* and *Helichrysum italicum* oils compared to nystatin, a common antimycotic drug. Furthermore, *C. zeylanicum*, *T. herba-barona* and *T. capitata* oils displayed the best fungicidal and sporicidal action, and their potential use in controlling infection in apiaries should be taken into consideration. Although previous in vitro studies have focused mainly on the fungicidal and non-sporicidal effects of essential oils, the observed sporicidal effect is very important because it suggests that some oils could be employed in the disinfection of combs or beekeeping equipment.

The antifungal activity of the *Thymus vulgaris* essential oil against *A. apis* was tested in previous studies using the agar dilution method [28,38]. Subsequently, *T. vulgaris* was confirmed to be a strong inhibitor of mycelium growth in the vapor phase as well [12,23]. This bioactivity is attributable to two phenolic monoterpenes, thymol and carvacrol, which are major bioactive components of *Thymus* oil [31]. This was confirmed in our bioassays, where carvacrol was identified as the major component of *Thymus capitatus* oil (68%) and *Thymus herba-barona* oil (60%), although thymol was present in small amounts.

Several products based on thymol are already commercially available against the ectoparasite mite *Varroa destructor* and are used inside hives [39]. An important advantage of beehive treatments with thymol is that no maximum residue limit in honey is imposed for this compound and no mite resistance against it has been found yet [39]. Furthermore, as it is highly volatile and apolar, thymol has a low persistence in honey compared to wax [39]. However, in bee wax, thymol residues can persist for several months [40]. In addition, thymol has negative effects on colony expansion, as it reduces the bee brood [39] and can affect phototaxis in treated bees but has no effects on colony mortality [41].

Our results confirm the excellent antimicrobic properties of *Cinnamomum* oil, already successfully applied in vitro and in vivo against *Paenibacillus larvae* [35,42,43,44], the ethiological agent of the american foulbrood, and against *A. apis* using both contact-dependent and contact-independent methods [30]. E-cinnamaldehyde was the major component present in the *Cinnamomum* oil used in our bioassays (79.3%). In addition to both thymol and carvacrol, E-cinnamaldehyde and other compounds (citral, citronellal, geraniol, eugenol and borneol) have been reported to have significant inhibitory effects on fungal growth [22,23,30,45]. Because honey bees produce secretions containing geraniol, citral, geranic and nerolic acids through the Nasonov glands, the use of these natural substances that are already present in the nest may not disturb the colony [46,47,48]. Moreover, many of these volatile and extractable compounds detected in the analyzed essential oils can be found in wax [49], honey [50] and in resins collected from plants and used to produce propolis by bees [51,52]. Therefore, it is plausible to expect an endogenous inhibitory action of all these compounds characteristic of the hive environment on this pathogen. In support of this hypothesis, we know that the presence of some pathogens and/or parasites, including *A. apis*, in the hive triggers an increase in the resin collection by foragers [53,54]. However, toxicological studies on the lethal or sublethal effects of essential oils on treated bees are very few. The studies by Eguaras et al. [28] and Ruffinengo et al. [25] did not show toxicity (mortality) in bees treated with *Tagetes minuta* and *Cinnamomun* oils, respectively. These results are encouraging and future studies on the application and effects of these essential oils in apiaries are needed.

Studies like ours underline the importance of seeking more natural products such as essential oils to counteract beehive diseases, because they reduce the risk of the contamination of bee products and resistance phenomena due to pathogens or parasites. Furthermore, the high volatility of essential oils makes them suitable for application in closed environments, such as hives. Finally, natural pesticides are biodegradable compared to the long persistence of synthetic pesticides in the environment [55].

Natural antibiotics based on essential oils may represent an alternative to chemically synthesized antibiotics. However, the transition from laboratory results to the application of a commercial formulation in the field is difficult for several reasons—the variable availability of the plant material, the variability of the oil from the same plant species due to different geographical origins or strains, and the different extraction or application methods used [56].

In conclusion, our in vitro study showed promising results regarding the use of essential oils from Mediterranean garrigue plants, especially *T. herba-barona* and *T. capitata,* and commercial *Cinnamomum zeylanicum* oil in the control of *Ascosphaera apis*. The advantages and disadvantages of the potential use of essential oils were also analyzed, and these require further elucidation. Therefore, it would be useful to conduct in vivo experiments in order to perform an in-depth evaluation of the effects of these oils on bees and on bee product residues.

## Figures and Tables

**Table 1:** Inhibition halo diameter (mean ± SD) of pure essential oils on *Ascosphaera apis* using the paper disc agar diffusion assay.

Essential Oils	Inhibition Halo Diameter (mm)
*Thymus herba-barona*	No growth
*Thymus capitatus*	No growth
*Helichrysum italicum*	40.0 ± 2.00 a ^1^
*Cinnamomum zeylanicum* (commercial)	24.8 ± 0.76 b
*Rosmarinus officinalis* (commercial)	16.0 ± 1.32 c
*Eucalyptus globulus*	10.0 ± 1.73 d
*Rosmarinus officinalis*	9.0 ± 1.00 d
*Myrtus communis*	8.0 ± 0.50 d
*Salvia desoleana*	No inhibition
*Salvia officinalis*	No inhibition
Nystatin	17.0 ± 1.32 c
Control	No inhibition

^1^ Means followed by the same letter are significantly different (ANOVA, followed by Tukey post-hoc test, *p* < 0.01).

**Table 2:** Minimum inhibitory concentration (MIC), minimum fungicidal concentration (MFC) and minimum sporicidal concentration (MSC) values of the four pure essential oils that showed the highest activity against *Ascosphaera apis* in the preliminary agar diffusion tests.

Essential Oils	MIC ^1^ (µg/mL)	MFC ^2^ (µg/mL)	MSC ^2^ (µg/mL)
*Thymus capitatus*	100 ^3^	300	400
*Thymus herba-barona*	100	300	400
*Cinnamomum zeylanicum*	200	200	400
*Helichrysum italicum*	˗	˗	500

^1^ Bioassay performed in Sabouraud broth. ^2^ Bioassay performed in Sabouraud dextrose agar. ^3^ Values of MIC, MFC and MSC are reported without measures of variability because they were homogeneous in all three replicates.

**Table 3:** Chemical characterization (% *w/w*) of the essential oils that showed the highest activity against *Ascosphaera apis* by means of gas chromatography–flame ionization detector (GC/FID) and gas chromatography–mass spectrometry analysis (GC/MS).

Components	CAS Number	r.t. ^α^ min	Essential Oils			
			*Thymus* *capitatus*	*Thymus* *herba-barona*	*Cinnamomun* *zeylanicum*	*Helichrysum* *italicum*
unknown		5.25	˗ ^β^	1.95	˗	0.56
α-Thujene	2867-05-2	5.76	1.60	1.00	1.1	˗
α-Pinene	7785-26-4	6.13	0.72	0.31	1.8	0.44
Camphene	79-92-5	7.17	0.22	0.50	0.2	0.27
Benzaldehyde	100-52-7	7.30	˗	˗	0.2	˗
Sabinene	3387-41-5	7.50	˗	˗	˗	˗
β-Pinene	127-91-3	7.88	0.08	2.23	0.1	0.20
Δ³-Carene	13466-78-9	9.10	1.54	˗	˗	˗
β-Mircene	123-35-3	9.14	2.11	1.05	-	0.27
α-Phellandrene	2243-33-6	9.30	0.10	0.18	0.1	0.19
α-Terpinene	99-86-5	9.57	n.d. ^γ^	1.04	˗	˗
Limonene	138-86-3	10.28	0.97	˗	0.2	1.71
p-Cymene	99-87-6	10.34	6.17	6.16	0.2	0.15
1.8-Cineolo	470-82-6	10.71	˗	0.96	˗	0.83
γ-Terpinene	99-85-4	11.80	6.33	4.49	˗	0.13
Terpinolene	586-62-9	12.75	1.15	0.20	˗	˗
Cinnamic aldehyde	104-55-2	14.72	˗	˗	79.3	˗
Terpinil acetate	80-26-2	22.53	˗	˗	-	0.39
Geranyl acetate	105-87-3	22.70	˗	˗	-	0.13
Camphor	76-22-2	23.59	˗	˗	0.1	˗
Linalool	78-70-6	23.71	n.d.	1.96	0.5	5.34
3-Octanol	589-98-0	23.82	0.32	˗	˗	˗
α-Thujone	546-80-5	23.98	˗	˗	-	˗
β-Thujone	471-15-8	24.10	˗	˗	-	˗
Bornyl acetate	5655-61-8	24.39	0.04	˗	0.5	˗
Farnesol	4602-84-0	24.45	˗	˗	0.2	˗
Linalyl acetate	115-95-7	24.51	˗	˗	˗	1.47
β-Caryophyllene	87-44-5	24.66	5.20	2.04	0.1	0.44
Linalyl isobutirrate	78-35-3	25.21	˗	˗	0.1	˗
Terpinen-4-ol	562-74-3	25.52	0.22	1.26	˗	˗
γ-Curcumene	28976-68-3	27.50	˗	˗	˗	2.05
Curcumene	644-30-4	27.65	˗	˗	˗	6.23
α-Terpineol	98-55-5	28.89	0.07	0.13	0.2	1.58
Borneol	464-45-9	29.11	n.d.	4.25	0.2	˗
Verbenone	1196-01-6	29.60	˗	˗	-	˗
β-Bisabolene	495-61-4	26.93	˗	˗	-	˗
Carvone	2244-16-8	30.13	˗	0.84	˗	˗
Nerol	106-25-2	32.65	˗	˗	˗	8.22
Neryl acetate	141-12-8	33.89	˗	˗	˗	51.59
Geraniol	106-24-1	34.27	˗	˗	0.2	˗
Caryophyllene oxide	1139-30-6	38.41	0.22	1.23	˗	˗
Metileugenol	95-15-2	39.44	˗	˗	0.3	˗
Neryl propionate	105-91-9	40.12	˗	˗	˗	5.51
Eugenol	97-53-0	41.01	˗	˗	11.9	˗
β-Eudesmol	473-15-4	42.15	˗	˗	˗	2.27
α-Eudesmol	473-16-5	42.89	˗	˗	˗	1.06
Cinnamyl alcohol	104-54-1	44.03	˗	˗	0.5	˗
Thymol	89-83-8	44.59	0.38	1.48	˗	0.16
Carvacrol	499-75-2	45.47	68.01	60.04	˗	0.13

^α^ retention time. ^β^ “-“ indicates that the compound was searched for but not detected in the EO. ^γ^ “n.d.” indicates that the compound was searched for and detected but was below the quantification limit.

## Data Availability

Derived data supporting the findings of this study are available from the corresponding author [AS] on request.
